# Isolation and Identification of a Novel Anti-Dry Eye Peptide from Tilapia Skin Peptides Based on In Silico, In Vitro, and In Vivo Approaches

**DOI:** 10.3390/ijms241612772

**Published:** 2023-08-14

**Authors:** Jian Zeng, Cuixian Lin, Shilin Zhang, Haowen Yin, Kaishu Deng, Zhiyou Yang, Yongping Zhang, You Liu, Chuanyin Hu, Yun-Tao Zhao

**Affiliations:** 1 College of Food Science and Technology, Modern Biochemistry Experimental Center, Guangdong Ocean University, Guangdong Province Engineering Laboratory for Marine Biological Products, Guangdong Provincial Key Laboratory of Aquatic Product Processing and Safety, Zhanjiang 524088, China; 2 College of Food Science and Engineering, Ocean University of China, Yu-Shan Road, Qingdao 266003, China; 3 Department of Biology, Guangdong Medical University, Zhanjiang 524023, China

**Keywords:** dry eye disease, tilapia skin peptides, isolation, molecular docking, in silico methods

## Abstract

Tilapia skin is a great source of collagen. Here, we aimed to isolate and identify the peptides responsible for combating dry eye disease (DED) in tilapia skin peptides (TSP). In vitro cell DED model was used to screen anti-DED peptides from TSP via Sephadex G-25 chromatography, LC/MS/MS, and in silico methods. The anti-DED activity of the screened peptide was further verified in the mice DED model. TSP was divided into five fractions (TSP-I, TSP-II, TSP-III, TSP-IV, and TSP-V), and TSP-II exerted an effective effect for anti-DED. A total of 131 peptides were identified using LC/MS/MS in TSP-II, and NGGPSGPR (NGG) was screened as a potential anti-DED fragment in TSP-II via in silico methods. In vitro, NGG restored cell viability and inhibited the expression level of Cyclooxygenase-2 (COX-2) protein in Human corneal epithelial cells (HCECs) induced by NaCl. In vivo, NGG increased tear production, decreased tear ferning score, prevented corneal epithelial thinning, alleviated conjunctival goblet cell loss, and inhibited the apoptosis of corneal epithelial cells in DED mice. Overall, NGG, as an anti-DED peptide, was successfully identified from TSP, and it may be devoted to functional food ingredients or medicine for DED.

## 1. Introduction

Dry eye disease (DED), a complicated ocular surface disease, impairs the health of millions of people around the world [1]. Many studies have shown that unhealthy lifestyles, autoimmune diseases, sleep deprivation, nutritional deficiencies, and high-fat diets increase the risk of DED [2,3]. Previous epidemiological data indicate that the prevalence of DED is much higher in the older than in the younger, especially among menopausal women [4,5]. Recently, the prevalence of DED has increased due to the popularity of video terminals [6]. Reportedly, seeking optical care for DED is the most common reason for outpatient eye clinics [7]. DED impairs the patient’s productivity in addition to producing eye irritation and other symptoms, resulting in considerable health expenditures and financial burdens, both directly and indirectly [8].

The causes and mechanisms of DED are complex and varied. Regardless of the etiology of DED, inflammation is the core pathological mechanism of DED. Tear hyperosmolarity is a potent stressor of DED, which can increase the expression of pro-inflammatory factors (e.g., cyclooxygenase-2 (COX-2), interleukin-1 (IL-1), tumor necrosis factor-alpha (TNF-α), etc.) in the ocular surface tissues and induce apoptosis of corneal epithelial cells [9]. This results in the overexpression of matrix metallopeptidase-9, destroying corneal barrier function. Therefore, the regulation of immune response is regarded as an important means to manage DED [10]. Today, many anti-inflammatory drugs are used in the clinical management of DED. For example, corticosteroids, cyclosporine, and lifitegrast [11]. They all act in various ways to suppress inflammatory signals on the ocular surface and break the inflammatory cycle, which assists DED patients in alleviating clinical symptoms. The long-term administration of them, however, can also lead to stinging, irritation on the eye’s surface, higher ocular pressure, and cataract development in the patient [12]. Recently, the role of bioactive peptides in the promotion of human health has gained importance [13]. Many biological activities of bioactive peptides, such as anti-inflammatory, antioxidant, anti-tumor, etc., have been widely reported [14,15,16]. Tsung-Chuan Ho et al. reported that pigment epithelium-derived factor-derived short peptide 29-mer has the function of improving DED [17]. Hence, it is of great potential to find bioactive peptides to combat DED.

Collagen is an essential component of skin, and it accounts for more than 70% of the dry weight of human skin [18]. Recently, the potential health benefits of collagen peptides have been a hot topic for researchers to explore. It is reported that tilapia skin contains about 30% collagen, and many bioactivities of tilapia skin collagen peptides have been wildly reported [19]. For example, Li et al. demonstrated that tilapia skin collagen peptides improved hepatic and renal injury induced by D-galactose [20]. Zhao et al. observed that tilapia skin peptides (TSP) improve premature ovarian failure and depressive and anxiety-like behavior [21,22]. In our previous study, TSP exerts anti-DED function in vivo and in vitro DED models [23]. However, specific active peptides with anti-DED activity in TSP have not been identified.

To overcome this scientific issue, this study aimed to screen potential anti-DED peptides from TSP via in silico strategy. Meanwhile, the special peptides with anti-DED function in TSP were further verified in in vivo and in vitro DED models.

## 2. Results and Discussion

### 2.1. Effects of TSP on Cell Viability of HCECs

**Figure 1:**
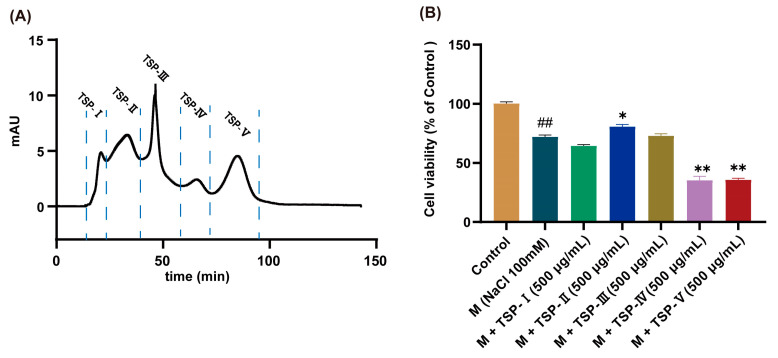
Results of the gel separation of tilapia skin peptides (TSP) and the anti-dry eye disease (DED) activities determination of each fraction. (**A**) Chromatogram of TSP isolated via Sephadex G-25 chromatographic column; (**B**) Results of anti-DED activities of each fraction of TSP, *n* = 5. ^##^ *p* < 0.01 vs. control group; * *p* < 0.05, ** *p* < 0.01 vs. M group.

TSP was isolated into five fractions via Sephadex G-25 column, which were named TSP-Ⅰ, TSP-Ⅱ, TSP-Ⅲ, TSP-Ⅳ, and TSP-V, respectively (Figure 1A). The anti-DED activities of the five fractions were further evaluated using the MTT approach. The results illustrated that the cell viability of HCECs exposed to NaCl (100 mM) was significantly reduced (*p* < 0.01 vs. control group, Figure 1B). At the same concentration (500 μg/mL), the cell viability of HCECs treated by TSP-Ⅱ significantly higher than that of the M group (*p* < 0.05, Figure 1B). TSP-Ⅰ and TSP-Ⅲ exerted no obvious influence on the cell viability of HCECs exposed to NaCl stress (*p* > 0.05, Figure 1B). After TSP-Ⅳ and TSP-V intervention, the cell viability of HCECs was obviously lower than that of the M group (both *p* < 0.01, Figure 1B). These data implied that the TSP-II had the strongest anti-DED activity (Figure 1B). Therefore, TSP-II was selected for further identification to discover peptides that were responsible for combating DED.

### 2.2. Identification of Peptides from TSP- II via LC/MS/MS

Amino acid sequences of TSP-II were identified via LC-MS/MS. The total ion flow chromatogram of TSP-II is shown in Figure S1A. The mass spectral data of TSP-II were further analyzed using PEAKS studio 8.0 software, and a total of 131 peptides were identified (Table S1). As illustrated in Figure S1B,C, the maximum and the minimum molecular weights of TSP-II were 1927 Da and 634 Da, respectively. The molecular weights of TSP-II were mainly distributed, ranging from 634 Da to 1500 Da (96.44%, Figure S1C). The number of peptides comprising TSP-II varied from 5 to 20 amino acids (Figure S1D). The most common peptides in TSP-II consisted of 9 and 11 amino acids, respectively (Figure S1D). There were 17 peptides consisting of 9 amino acids and 16 peptides consisting of 11 amino acids in TSP-II (Figure S1D). The peptides consisting of 5, 17, and 20 amino acids, respectively, were the least prevalent in TSP-II (Figure S1D). The characterization of bioactive peptides with molecular weights < 1500 Da was reported in many studies [24]. Most of the peptides of TSP-II were consistent with this feature. Thus, the anti-DED peptides may be present in a group of peptides with molecular weights less than 1500 Da in TSP-II.

### 2.3. Screening of Bioactive Peptides via ToxinPred and PeptideRanker Web Servers

To screen the potential anti-DED peptides, ToxinPred was used to predict the toxin of all identified peptides. GPMGPRGPGPPPGSSGPQ was a potent toxic peptide predicted using ToxinPred (Table S1). Then, PeptideRanker was used to analyze the bioactivity probabilities of the remaining 130 non-toxic peptides. The results were displayed as PeptideRanker scores, from 0 to 1. The higher the PeptideRanker score, the higher the likelihood for a peptide to be bioactive [25]. The threshold of the PeptideRanker score was set to 0.6, and 53 potent anti-DED peptides were selected for subsequent screening (Table S2). It is a vast group of active peptides in which potential anti-DED active peptides may be hidden.

### 2.4. Screening of AIPs and Allergenicity Prediction

Inflammation is recognized as an important part of the pathological mechanism of DED and has been seen as an indicator to evaluate the severity of DED [26]. Therefore, peptides with anti-inflammatory properties may be excellent lead compounds to combat DED. Nevertheless, it is a long process to screen AIPs via laboratory means. High costs of experimentation and huge investments in human resources are often not commensurate with the expected benefits. In silico tools offer an excellent solution to overcome this challenge. AIPpred is one of the effective tools for predicting AIPs among bioinformatics tools [27]. Wongsrangsap, N et al. reported shorter peptide chains show better bioactivity than longer ones [28]. And the longer the peptides, the higher the cost will be spent in commercial applications in the future [29]. All the above reasons were taken into consideration, and the peptides of less than 11 amino acids were selected for further screening. There were 19 candidate peptides with amino acid numbers less than 11 of the 53 bioactive peptides (Table S3). Then, 17 peptides were predicted to be AIPs using AIPpred from the 19 bioactive peptides (Table S3). DFLLK achieved the highest AIP score (0.6023) and may be a potentially efficient candidate for the treatment of DED. Additionally, allergy is a common challenge faced by many foods and medicines, and it seriously affects human health [30]. To avoid the hazards caused by allergic peptides and ensure food and pharmaceutical security, AllerTOP v. 2.0 was used to predict the allergy of the 17 AIPs. Only three AIPs were determined to be non-allergenic peptides using AllerTOP v. 2.0 (Table S3).

The amino acid sequence characteristics of the three candidates were further analyzed and revealed that they matched the characteristics of some AIPs. The presence of positively charged and hydrophobic amino acids at the N- and C-termini of peptides is assumed to assist in anti-inflammatory responses [31]. For example, Arg (R)-containing peptides at the C-terminus, YGIYPR and LDAVNR, exhibited anti-inflammatory properties [32,33]. The AIPs screened in this work, DFCPPGFNTK (DFC), EAPDPLRN (EAP), and NGGPSGPR (NGG), all fitted this profile. Notably, GPR, an identified anti-inflammatory peptide, was found in NGG [34]. Polar groups observed in AIPs are mainly found at the C-terminals [31]. NTK, at the C-terminus of DFC, belongs to polar amino acids.

So, DFC, EAP, and NGG were selected for further investigation according to toxicity prediction, bioactivity score, amino acid sequence analysis, and anti-inflammatory and allergic properties.

### 2.5. Screening Anti-DED Peptides by Molecule Docking with COX-2

To further screen the anti-DED peptides, molecular docking was carried out. Molecular docking is an approach for screening bioactive ingredients that are based on the 3D structure of target proteins. It investigates the intermolecular interactions, predicts their binding patterns, and predicts the affinity and activity of small molecules.

COX-2, a popular target in pharmaceutical design, is mainly involved in prostaglandin production and promotes the inflammatory response [35]. The inhibition of COX-2 activity blocks the formation of pro-inflammatory mediators, thereby alleviating ocular surface inflammation [36]. Ji et al. indicated that COX-2 is a potential molecular target for the treatment of DED [37]. Studies have reported that some bioactive peptides exhibit anti-inflammatory activity by modulating the expression of COX-2 [31]. Our previous study also demonstrated that TSP suppresses the protein expression level of COX-2 in NaCl-stimulated HCECs [23]. Therefore, it is possible that peptides targeted by COX-2 may have potential anti-DED functions.

HPEPDOCK serve is an efficient molecular docking platform. Yang et al., screen monoamine oxidase A inhibitory peptides utilizing HPEPDOCK and other active peptide screening tools successfully [38]. Peptides screened via HPEPDOCK exert protective effects on oxidative stress-induced ovarian granulosa cell damage [39]. In this present study, HPEPDOCK serve was employed to carry out molecular docking assay. On the HPEPDOCK server, each peptide is docked with COX-2 100 times, and the optimal docking pose will have the lowest HPEPDOCK score. A lower score indicates that the peptide binds more tightly to COX-2 and may have higher anti-DED activity. Among the three peptides, NGG exhibited the highest HPEPDOCK score (227.219), followed by DFC (189.871) (Table 1). EAP’s HPEPDOCK score (−139.07) was superior to that of NGG (Table 1). Therefore, NGG may be a powerful candidate for the prevention and treatment of DED. It is worth noting that NGG, DFC, and EPA could not be found in the Uniprot and BIOPEP-UWM (Table 1). Therefore, they can be defined as novel peptides.

### 2.6. Assay of In Vitro Anti-DED Activity of NGG

**Figure 2:**
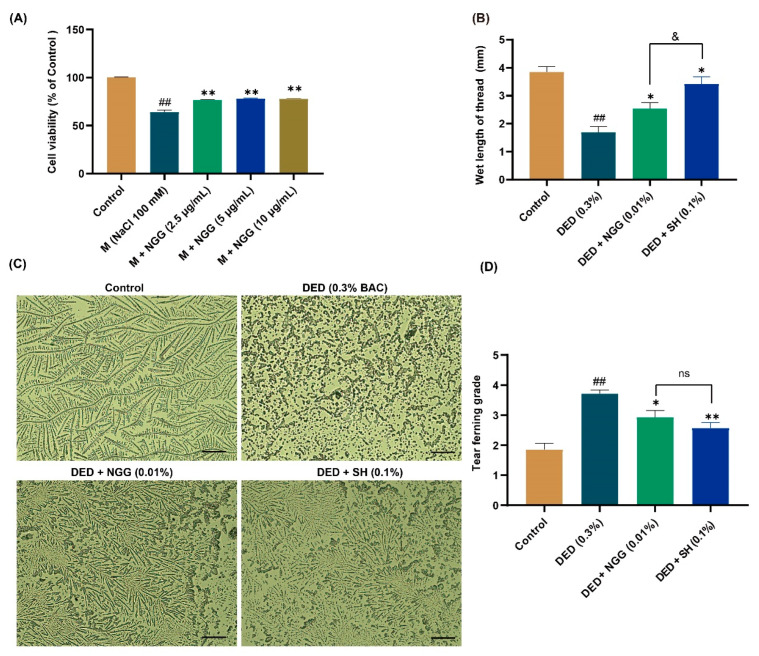
Results of anti-DED activity of NGGPSGPR (NGG) in vitro and in vivo. (**A**) Effect of NGG on cell viability of NaCl-induced HCECs, *n* = 5, **^##^** *p* < 0.01 vs. control group; ** *p* < 0.01 vs. M group. (**B**) Results of tear secretion in mice, *n* = 15 eyes, **^##^** *p* < 0.01 vs. control group; * *p* < 0.05 vs. DED group; ^&^ *p* < 0.05 vs. DED + SH group. (**C**) Representative images of tear fern-like crystals in mice, scale bar = 50 μm. (**D**) Results of tear ferning grade in mice, *n* = 14 eyes. **^##^** *p* < 0.01 vs. control group; * *p* < 0.05, ** *p* < 0.01 vs. DED group; ns, *p* > 0.05 vs DED + SH group.

To verify our speculation about the anti-DED activity of NGG, NGG was synthesized. The sequence information of the synthesized NGG was examined via LC/MS/MS. The result showed that the structural information of the synthesized NGG was correct (Figure S2). MTT assay assayed that NGG at doses of 6.25 μg/mL and 12.5 μg/mL were not toxic to HCECs (both *p* > 0.05 vs. control group, Figure S3). NGG (25 μg/mL) significantly decreased the cell viability of HCECs compared with that of the control group (*p* < 0.01, Figure S3). The anti-DED effect of NGG on the in vitro DED model of HCECs is shown in Figure 2A. Moreover, 100 mM NaCl obviously reduced the cell viability of HCECs (*p* < 0.01 vs. control group, Figure 2A). After treatment with NGG (2.5 μg/mL, 5 μg/mL, and 10 μg/mL), the viabilities of HCECs were obviously restored compared with that of the M group (both *p* < 0.01, Figure 2A). Those results indicated that NGG showed good anti-DED activity.

### 2.7. Effects of NGG on Tear Production and Tear Ferning in DED Mice

Tears are a fluid membrane structure consisting of an aqueous layer, a mucin layer, and a lipid layer that covers the surface of the eyeball, also named tear film [40]. Healthy tear film delivers nutrients to the cells of the ocular surface, maintains surface wetness, and reduces rubbing damage to the cornea and conjunctiva [41]. Reduction in tear production is an essential clinical feature of DED, and ophthalmologists aim to restore ocular surface homeostasis by improving tear secretion function in the treatment of DED [42,43]. Earlier studies reported that tear production in DED mice is significantly lower than that of healthy mice [36,44]. In this study, the reddening length of the phenol red cotton threads in the DED group was substantially shorter than that of the control group (*p* < 0.01, Figure 2B). In contrast, it was much longer in the DED + NGG and DED + SH groups than that in the DED group (both *p* < 0.05, Figure 2B). These data implied that BAC induction impaired the tear secretion function of mice, while NGG and SH intervention improved it. The length of the reddening of phenol red cotton threads in the DED + NGG group was statistically shorter than that of the DED + SH group (*p* < 0.05, Figure 2B).

Reduction in tear secretion results in the disruption of the balance of the ocular surface microenvironment [42]. An obvious indicator of that is the lack of mucin in sufficient quantities in the tear film, which manifests clinically via insufficient formation of tear fern-like crystals [45]. Additionally, depressed tear production results in the development of ocular surface inflammation, which will further exacerbate DED symptoms [40]. In the control group, the tear fern-like crystals were formally complete, while in the DED group, no fern-like crystals were observed (Figure 2C). After the intervention of NGG and SH, the shapes of the tear fern-like crystals were clearly restored (Figure 2C). Statistically, the grade of tear fern-like crystals in the DED group was significantly higher than that of the control group (*p* < 0.01, Figure 2D). The grades of tear fern-like crystals in the DED + NGG and DED + SH groups were significantly lower than that of the DED group (*p* < 0.05, *p* < 0.01, Figure 2D). The data showed that there were no obvious differences in restoring tear ferning between the DED + NGG group and the DED + SH group (*p* > 0.05, Figure 2D). The results of the tear ferning test indicated that BAC disrupted tear film homeostasis in mice, while the balance of tear film homeostasis was improved after NGG or SH intervention.

### 2.8. Effects of NGG on Conjunctival Goblet Cells (CGCs) in DED Mice

**Figure 3:**
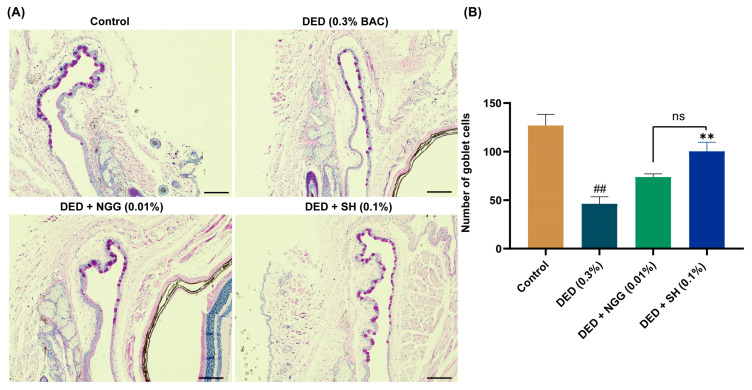
Effects of NGG on conjunctival goblet cells (CGCs) in BAC-induced DED mice. (**A**) Representative images of PAS staining in mice of each group, scale bar: 50 μm; (**B**) Results of NGG on the number of CGCs in BAC-induced DED mice, *n* = 5 eyes. ^##^ *p* < 0.01 vs. control group; ** *p* < 0.01 vs. DED group; ns, *p* > 0.05 vs DED + SH group.

Effects of NGG on CGCs in DED mice were evaluated via PAS staining (Figure 3A). The loss of CGCs has been widely recognized as one of the important pathological changes in DED [46]. Previous research demonstrated that the number of CGCs in DED mice is markedly lower than that of healthy mice [43,47]. Here, in the DED group, BAC clearly leads to the absence of CGCs, and the number of CGCs was significantly lower than that of the control group (*p* < 0.05, Figure 3A,B). The main physiological function of CGCs is to secrete mucins. There are a variety of mucins on the ocular surface, which are significant for maintaining the homeostasis of the ocular surface microenvironment. Lubricating, removing ocular surface debris, and protecting epithelial tissue are the main functions of mucins [48]. In recent years, immunomodulatory effects of mucin have also been noted, and they have made a considerable contribution to the maintenance of ocular surface health [49]. Other evidence suggests that CGC loss is strongly associated with chronic inflammation and apoptosis in the ocular surface, and the loss of CGCs leads to further instability of the tear film [50,51]. After intervention with NGG or SH, the number of CGCs was greater than that of the DED mice (*p* > 0.05, *p* < 0.01, Figure 3B). There was no statistical difference in the prevention of CGC loss between the DED + NGG group and the DED + SH group (*p* > 0.05, Figure 3B). Those results proved that NGG exhibited the effect of preventing the destruction of CGCs via BAC.

### 2.9. Effects of NGG on Corneal Epithelium in DED Mice

**Figure 4:**
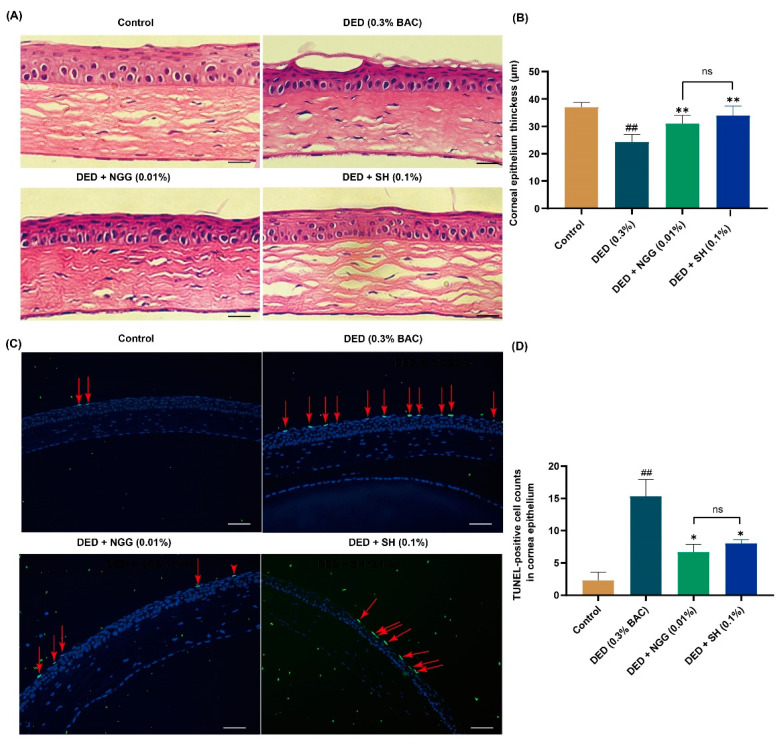
Effects of NGG on corneal epithelium in BAC-induced DED mice. (**A**) Representative images of H&E staining of corneas in mice of each group, scale bar: 50 μm. (**B**) Results of NGG on the thickness of corneal epithelial cells in BAC-induced DED mice, *n* = 9 eyes, **^##^** *p* < 0.01 vs. control group; ** *p* < 0.01 vs. DED group; ns, *p* > 0.05 vs DED + SH group. (**C**) Representative images of TUNEL-positive cells in mice corneal epithelium, and the red arrows point to the apoptotic corneal epithelial cells in the images, scale bar: 50 μm. (**D**) Results of NGG on the number of TUNEL-label corneal epithelial cells in BAC-induced DED mice, *n* = 3 eyes. **^##^** *p* < 0.01 vs. control group; * *p* < 0.05 vs. DED group; ns, *p* > 0.05 vs DED + SH group.

An additional critical pathological finding in DED is corneal lesions. Corneal damages in patients with DED include the thinning of the epithelial cell layer and apoptosis. It is a complex physiological event in which tear osmolarity stimulation and inflammation are involved. The cornea is an organ that is in direct contact with the external environment and is also wetted using the tear film. Both external environmental stimuli and changes in tear film cause corneal lesions. In Figure 4A,B, the corneal epithelial thickness of the DED group was significantly thinner than that of the control group (*p* < 0.01). These data are consistent with previous findings [43,47]. After intervention with NGG and SH, the corneal epithelial thicknesses were significantly higher than that of the DED group (both *p* < 0.01, Figure 4A,B). There was no meaningful difference in corneal epithelial thickness between the DED + NGG group and DED + SH group (*p* > 0.05, Figure 4B). Apoptosis contributes to the onset and progression of DED. Growing pieces of evidence have reported that the number of apoptotic corneal epithelial cells in DED mice increases significantly [44,47]. In Figure 4C and D, the number of TUNEL-labeled epithelial cells in the DED group was obviously increased compared with that of the control group (*p* < 0.01). After intervention with NGG and SH, the number of TUNEL-labeled epithelial cells in the DED + NGG and NGG + SH groups was significantly decreased compared with that of the DED group (both *p* < 0.05, Figure 4C,D). There was no meaningful difference in the number of TUNEL-labeled epithelial cells between the DED + NGG group and the DED + SH group (*p* > 0.05, Figure 4B). These results demonstrated that NGG possessed a role in improving corneal epithelial physiology. Molecular docking results showed that NGG may have anti-inflammatory properties. So, NGG may also improve corneal lesions through its anti-inflammatory activity. That may be another plausible explanation for the inhibition of corneal lesions via NGG.

### 2.10. Analysis of COX-2–NGG Interactions and Effects of NGG on COX-2 Protein Expression

**Figure 5:**
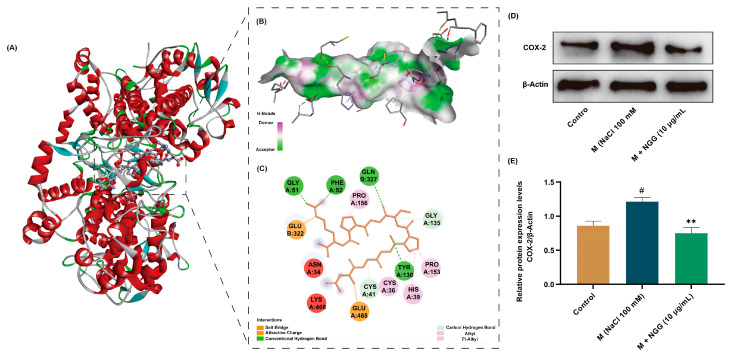
Interactions of COX-2-NGG and effects of NGG on COX-2 protein expression. (**A**) Predicted 3D structure of NGG and COX-2 complex from molecular docking. (**B**) Binding interactions of NGG and COX-2 with amino acid residues in 3D display. (**C**) Binding interactions of NGG and COX-2 with amino acid residues in 2D display. (**D**) Representative Western blot images of COX-2. (**E**) Statistical results of the Western blot results of COX-2, *n* = 4. ^#^ *p* < 0.05 vs. control group; ** *p* < 0.01 vs. M group.

Here, the docking pose of NGG with COX-2 was further analyzed to explore the mechanisms of their interactions. Theoretically, inhibitory peptides or other suppressing small molecules reduce or completely deactivate an enzyme by occupying or blocking the active sites of that enzyme [52]. In this present study, NGG stayed within the active pocket of COX-2 (Figure 5A), which probably was the structural basis for their action. Hydrogen bonding is considered an important force that maintains the stable conformation formed by small molecules and enzymes and positively affects the activity of substances [53]. Here, NGG formed four conventional bonds (Tyr A: 130, Gln B: 327, Gly A: 51, and Phe A: 52) and second unconventional hydrogen bonds (Gly A:35 and Cys A:41) with the active amino acid residues of COX-2 (Figure 5B,C). Furthermore, hydrophobic and electrostatic interactions also play important roles in the conformation of proteins. Two types of hydrophobic interactions and two kinds of electrostatic interactions were observed in the docking model of NGG with COX-2 (Figure 5B,C). All these interactions led to a conformational shift of COX-2, which may result in the potential inhibition of COX-2 via NGG. And NGG may have potential anti-inflammatory effects. The altered conformation of COX-2 may be a potential molecular basis for the role of NGG in improving DED.

The results of molecular docking implied that NGG exhibited anti-DED activity by suppressing the activity of COX-2. To further verify this result, the protein expression level of COX-2 was detected using Western blot (Figure 5D). The results showed that NaCl stimulation activated COX-2 in HCECs, resulting in its expression level being significantly higher than that of the control group (*p* < 0.05, Figure 5E). The result is consistent with the previously published article [54]. After treatment with NGG (10 μg/mL), the expression level of COX-2 was obviously lower than that of the M group (*p* < 0.01, Figure 5E). This finding supports the results of molecular docking. Therefore, NGG exhibited anti-inflammatory properties by inhibiting COX-2, which may be one of the molecular mechanisms via NGG improving DED.

## 3. Materials and Methods

### 3.1. Materials

Human corneal epithelial cells (HCECs) were purchased from Guangzhou Jennio Biotech Co., Ltd. (Guangzhou, China). Sodium Hyaluronate (SH, H20150150) eye drops were obtained from URSAPHARM Arzneimittel GmbH (Saarbrücken, Germany). Sephadex G-25 gel (S8141) was purchased from Beijing Solarbio Science & Technology Co., Ltd. (Beijing, China). Tetramethylazolium salt (MTT, S19063) was purchased from Shanghai Yuanye Biotechnology Co., Ltd. (Shanghai, China). Benzalkonium chloride (BAC, C12506740) was bought from Shanghai Macklin Biochemical Technology Co., Ltd. (Shanghai, China). Tear test phenol red cotton thread was obtained from Tianjin Jingming New Technological Development Co., Ltd. (Tianjin, China). The terminal deoxynucleotidyl transferase dUTP nick end labeling kit (TUNEL, E-CK-A321) was obtained from Elabscience Biotechnology Co., Ltd. (Wuhan, China). Periodic Acid-Schiff Staining Kit (PAS, C0142S) and hematoxylin and eosin staining Kit (H&E, C0105S) were obtained from Beyotime Biotechnology Co., Ltd. (Shanghai, China). Formic acid (FA, 695076), DL dithiothreitol (DTT, D0632), and iodoacetamide (IAA, I6125) were obtained from Sigma (St. Louis, MO, USA). Anti-cyclooxygenase-2 (COX-2, 12282S) and anti-β-Actin (4970) antibodies were obtained from Cell Signal Technology (Danvers, MA, USA). Modified eagle’s medium (11095080), fetal bovine serum (1907422), and Penicillin-Streptomycin solution (2289325) were obtained from Gibco (Grand Island, NY, USA). Immobilon™ Western Chemiluminescent HRP Substrate (WBKLS0100) and polyvinylidene fluoride (PVDF) membranes (IPVH00010) were obtained from Millipore (Bedford, MA, USA).

### 3.2. Preparation of TSP

TSP was prepared using the methodology described in our prior article [22]. Briefly, the tilapia skin was cut up and dehydrated with isopropyl alcohol. Tilapia skin was digested by neutral protease and alkaline protease enzymes. Then, the supernatant was collected after centrifugation of the enzymatic hydrolysis solution. The supernatant was filtered via a 10 kDa ultrafiltration membrane, and the filtrate was collected and lyophilized to obtain TSP. Molecular weights of TSP mainly concentrated in the range of 180~3000 Da (92.63%), which indicated that TSP consisted mainly of peptides with 2 to 27 amino acids [22].

### 3.3. Isolation of TSP and Screening of Its Active Fractions

TSP was isolated using Sephadex G-25 gel separation techniques. Briefly, TSP was dissolved in ultrapure water and filtered by 0.45 μm filters. The filtrate was separated with the Sephadex G-25 column (16 mm × 300 mm). Ultrapure water was used as the mobile phase, and TSP was eluted at a flow rate of 0.45 mL/min under the monitoring wavelength of 280 nm. The fractions corresponding to each peak were collected, lyophilized, and stored at −80 °C for following studies.

### 3.4. Peptides Sequence Identification of TSP via LC/MS/MS

The fraction of TSP isolated using Sephadex G-25 with anti-DED effect will be further identified via LC/MS/MS. LC/MS/MS analysis was performed with the Ultimate 3000 system (Thermo, Waltham, MA USA) and the Q Exactive^TM^ Hybrid QuadrupoleOrbitrap^TM^ Mass Spectrometer (Thermo, USA). Briefly, TSP was reduced and alkylated via DTT and IAA, respectively. Then, TSP was dissolved in 0.1% FA. And 5 μL TSP solution was injected into the reversed-phase ReproSil-Pur C18 column (150 μm × 150 mm, 1.9μm, Dr. Maisch, Germany). The mobile phase consisted of phase A (0.1% FA in water) and phase B (20% 0.1% FA in water 80% acetonitrile). TSP was eluted according to the following parameters: (1) Flow rate: 600 nL/min; (2) Gradient program: 0~2 min, 4~28% phase B; 2~245 min, 8~228% phase B; 45~255 min, 28~240% phase B; 55~256 min, 40~295% phase B; 56~266 min, 95% phase B. The following parameters were applied to Mass Spectrometer: (1) MS: MS resolution: 70,000 at 400 *m*/*z*; MS precursor *m*/*z* range: 300~1800. (2) MS/MS: Production scan range: start from *m*/*z* 100; Activation Type: HCD; Normalized Coll. Energy: 28; Activation Time: 66 ms. The mass spectrometry data were analyzed using PEAKS Studio 8.5 software (Bioinformatics Solutions, Waterloo, ON, Canada).

### 3.5. Screening the Bioactive Peptides via In Silico Methods

Toxicity of identified peptides was analyzed using ToxinPred (http://crdd.osdd.net/raghava/toxinpred/, accessed on 9 December 2021) [55]. The bioactivity probabilities of the non-toxic peptides were further predicted using PeptideRanker (http://distilldeep.ucd.ie/PeptideRanker/, accessed on 9 December 2021). AIPpred (http://www.thegleelab.org/AIPpred/) was used to seek anti-inflammatory peptides (AIPs) [27]. AllerTOP v. 2.0 (http://ddgpharmfac.net/AllergenFP/) was applied to predict the allergenicity of the peptides [56]. The aforementioned tools were accessed on 1~5 January 2022. Peptide sequences were uploaded to the UniProt (https://www.uniprot.org) and BIOPEP-UWM (https://biochemia.uwm.edu.pl/biopep/start_biopep.php) databases, and peptide not recorded in either database will be defined as a completely new one [38,57]. These two databases were accessed on 4 March 2023.

### 3.6. Molecular Docking and Peptide Chemical Synthesis

The protocol of molecular docking was designed according to the methodology in the published studies [38,58]. Briefly, molecular docking was carried out on the HPEPDOCK server (http://huanglab.phys.hust.edu.cn/hpepdock/, accessed from 28 February to 6 March 2022) [59]. The crystal structure of COX-2 (PDB ID: 5kir) was downloaded from the RCSB Protein Data Bank (https://www.rcsb.org/, accessed from 28 February 2022) [60]. The crystal structure of COX-2 (set as a ligand) and the sequence of the peptide (set as an acceptor) were uploaded to HPEPDOCK server. The best docking model has the highest negative HPEPDOCK score. The docking results were visualized and displayed using Discovery Studio Visualizer 2021 (Biovia 2021). The peptide with the highest negative HPEPDOCK score was synthesized by the Cellmano Biotech Limited Corporation (Hefei, China).

### 3.7. Cell Culture and Treatment

HCECs were cultured in modified Eagle medium supplemented with 10% fetal bovine serum and 1% antibiotics (penicillin/streptomycin). Cells were maintained in a humidified incubator at 37 °C and 5% CO_2_.

HCECs were seeded into 96-well plates to culture 24 h. Then, cells were treated with TSP or isolated and identified peptides for 12 h in the presence or absence of NaCl. After 12 h, the cell viability of HCECs was measured using MTT assay. Briefly, the medium was abandoned, and the cells were washed with phosphate buffer (PBS, pH 7.2) for 3 times. Then, 100 μL of MTT solution (0.5 mg/mL, dissolved in PBS) was added to each well and incubated at 37 °C for 4 h. The supernatant in each well was removed, and each well received 150 μL of dimethyl sulfoxide to dissolve the formazan. The results were obtained by measuring absorbance at 490 nm with a microplate reader (BioTek, Winooski, VT, USA).

### 3.8. Animals and Treatment

C57BL/6 male mice (aged 6–7 weeks) were obtained from Guangdong Medical Laboratory Animal Center (Guangzhou, China). Animal experimentation was carried out in strict accordance with the requirements of the Animal Ethics Committee of Guangdong Ocean University (approval number: 2022062101) and the Guidelines for the Care and Use of Laboratory Animals of Guangdong Ocean University.

Mice were randomly grouped into 4 groups (8 mice per group, *n* = 16 eyes): control group (dropped eyes with normal saline), DED group (dropped eyes with 0.3% BAC firstly, then normal saline was dropped eyes 1 h later), DED + NGG group (dropped eyes with 0.3% BAC firstly, then 0.01% NGG was dropped eyes 1 h later), and DED + SH group (dropped eyes with 0.3% BAC firstly, then SH was dropped eyes 1 h later), respectively. SH was used as a positive drug. The eye drop operation started at 8:30 am and 6:30 pm, respectively. Each eye was treated with 5 μL of each eye drop at a time, and the whole assay lasted for 14 days.

### 3.9. Tear Production and Tear Ferning Test

Tear production was measured as described in previous publications [61]. Briefly, mice were anesthetized, and phenol red cotton thread was inserted into the palpebral conjunctiva of the lower eyelid of the mice for 30 s to detect the length of the thread turning red. The length of the cotton thread turning red reflects the level of tear secretion in mice. Tear ferning test was assayed as follows [45]: tears of each eye were collected and spread on glass slides. The tear fern-like patterns were obtained under the microscope after drying at room temperature for 3 h, and the graphs were scored according to the method reported in the literature [45].

### 3.10. Tissue Preparation for Histological Assessment

After anesthetizing the mice, PBS and 4% paraformaldehyde were injected into the hearts of mice. The complete ocular surface tissue was excised and immersed in 4% paraformaldehyde for 24 h. Then, tissues were dehydrated and embedded in paraffin. Paraffin sections were prepared at a thickness of 4 μm.

### 3.11. H&E Staining and PAS Staining Analysis

Paraffin sections were used for H&E and PAS assays. All procedures were performed in strict line with the kit manufacturer’s instructions. The results of H&E staining were used to detect the thickness of the corneal epithelium, and PAS staining was performed to measure the number of conjunctiva goblet cells (CGCs). The central corneal thickness of each eye in three sections was calculated using Image J software, https://imagej.net/ij/ (NIH Image). The number of CGCs in the whole conjunctiva from each eye in three sections was counted using Image pro plus 6.0 software (Media Cybernetics).

### 3.12. In Situ TUNEL Staining

TUNEL test was utilized to measure the number of apoptotic cells in corneal epithelium of mice according to the manufacturer’s guidelines. Briefly, paraffin sections were dewaxed and rehydrated. For each sample, proteinase k (100 μL) was added and reacted at 37 °C for 20 min. Sections were washed with PBS and incubated with terminal deoxynucleotidyl transferase (TDT) equilibration buffer (100 μL) for 20 min at 37 °C. After aspiration of the TDT equilibrium, 50 μL of labeling working solution (TDT equilibration buffer:Label solution:TDT enzyme = 7:2:1, *v*/*v*/*v*) was added to each sample and incubated for 60 min in a dark chamber at 37 °C. The sections were washed 3 times with PBS. Apoptosis of corneal epithelial cells was examined via fluorescence microscopy (Leica, Wetzlar, Germany) after re-staining the nuclei with DAPI (4′,6-diamidino-2-phenylindole). The representative images of central corneal epithelium were obtained from each section under the fluorescence microscope, and the number of TUNEL-label cells on each picture was counted using Image J software (NIH Image). The number of TUNEL-positive cells in every eye was counted in three sections.

### 3.13. Western Blot

Proteins from HCECs were extracted using protein extraction reagent (RIPA lysis buffer: protease inhibitor: phosphatase inhibitor = 100:1:1, *v*/*v*/*v*). The protein concentration of the cell samples was detected according to the instructions of the BCA protein quantification kit. The protein samples (30 μg per sample) were separated in SDS-polyacrylamide gels, and the target proteins were transferred to the PVDF membranes. After soaking in 5% no-fat milk (dissolved into TTBS, *m*/*v*) for 2 h at room temperature, the membranes were incubated with the primary antibodies of COX-2 and β-Actin overnight at 4 °C, respectively. Then, the membranes were washed with TTBS (Tris Buffered Saline with Tween-20) and incubated with horseradish peroxidase-conjugated secondary antibody for 1 h at room temperature. The blots of target proteins were visualized using immobilon ^TM^ Western Chemiluminescent HRP Substrate. ChemiDoc ™ XRS ^+^ system (Bio-Rad, Hercules, CA, USA) was used to record the images of blots, and the expression levels of target proteins were measured using Image J software (NIH Image).

### 3.14. Statistical Analysis

All experimental data were analyzed using GraphPad Prism 8.0 (GraphPad Software, San Diego, USA). Data were shown as mean ± SEM. Marked differences (*p* < 0.05) between different experimental groups were determined using One-way ANOVA followed by Duncan’s multiple-range post hoc analysis.

## 4. Conclusions

In summary, the anti-DED active fragment-NGG in TSP was successfully traced via a combination of virtual screening and traditional experimental approaches, and the anti-DED activity of NGG was verified using in vitro and in vivo experiments. This study provides new ideas for the isolation and identification of food-derived bioactive peptides, and NGG is expected to be developed as a new product for the prevention and management of DED.

Nevertheless, the role of NGG in alleviating DED was explored in only two DED models. And the potential mechanism of NGG on DED has not been clarified completely. In our future studies, the effects and biological mechanisms of NGG to alleviate DED will be explored deeply in other DED models. In addition, the exploration of the structure-activity relationship of NGG is also a research point of our interest.

## Figures and Tables

**Table 1:** Results of TSP-II screened using HPEPDOCK server, AllerTOP v. 2.0, Uniprot and BIOPEP-UWM.

No.	Peptide	Length	HPEPDOCK Score	Allergenicity	Uniprot	BIOPEP-UWM
1	NGGPSGPR	8	−227.219	No	No	No
2	DFCPPGFNTK	10	−189.871	No	No	No
3	EAPDPLRN	8	−139.07	No	No	No

## Data Availability

The data presented in this study are available on request from the corresponding author.

## Supplementary Materials

The supporting information can be downloaded at: https://www.mdpi.com/article/10.3390/ijms241612772/s1.
