# Supplementary material for: Isolation and Identification of a Novel Anti-Dry Eye Peptide from Tilapia Skin Peptides Based on In Silico, In Vitro, and In Vivo Approaches
Source: Int J Mol Sci. 2023 Aug 14;24(16):12772. doi: 10.3390/ijms241612772 (PMC10454390; doi:10.3390/ijms241612772)
Supplement: Supplementary file 1 [file ijms-24-12772-s001.zip › ijms-2496094-supplementary.pdf]

Isolation and Identification of a Novel Anti-Dry Eye Peptide from Tilapia Skin Peptides Based on In Silico, In Vitro, and In Vivo Approaches

Jian Zeng [1], Cuixian Lin [1], Shilin Zhang [1], Haowen Yin [1,2], Kaishu Deng [1], Zhiyou Yang [1], Yongping Zhang [1], You Liu [1], Chuanyin Hu [3,*] and Yun-Tao Zhao [1,*]

1 College of Food Science and Technology, Modern Biochemistry Experimental Center, Guangdong Ocean University, Guangdong Province Engineering Laboratory for Marine Biological Products, Guangdong Provincial Key Laboratory of Aquatic Product Processing and Safety, Zhanjiang 524088, China
2 College of Food Science and Engineering, Ocean University of China, Yu-Shan Road, Qingdao 266003, China
3 Department of Biology, Guangdong Medical University, Zhanjiang, 524023, China
* Correspondence: huchuanyin@gdmu.edu.c or hucyin@126.com (C.H.); zhaoyt@gdou.edu.cn (Y.-T.Z.).

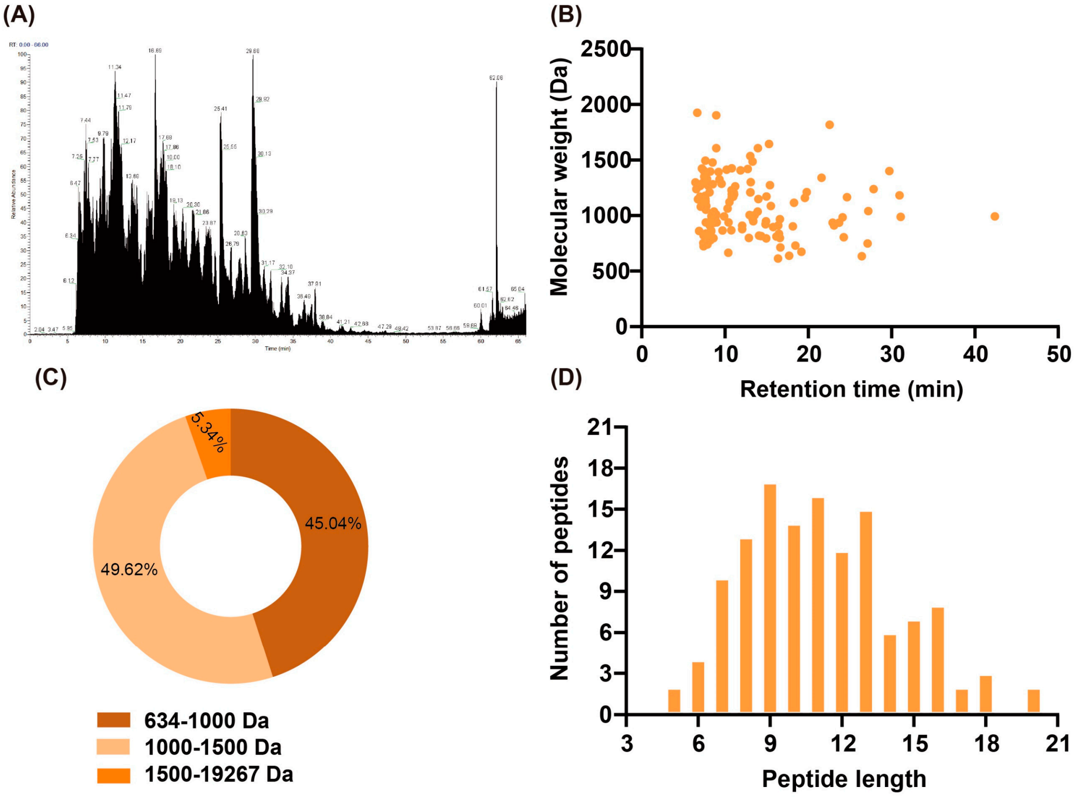

Figure. S1 Result of LC/MS/MS identification of tilapia skin peptides-II (TSP-II). (**A**) Total ion flow diagram of TSP-II; (**B**,**C**). Distribution of molecular weight of TSP-II. (**D**) Distribution of the length of amino acids for TSP-II.

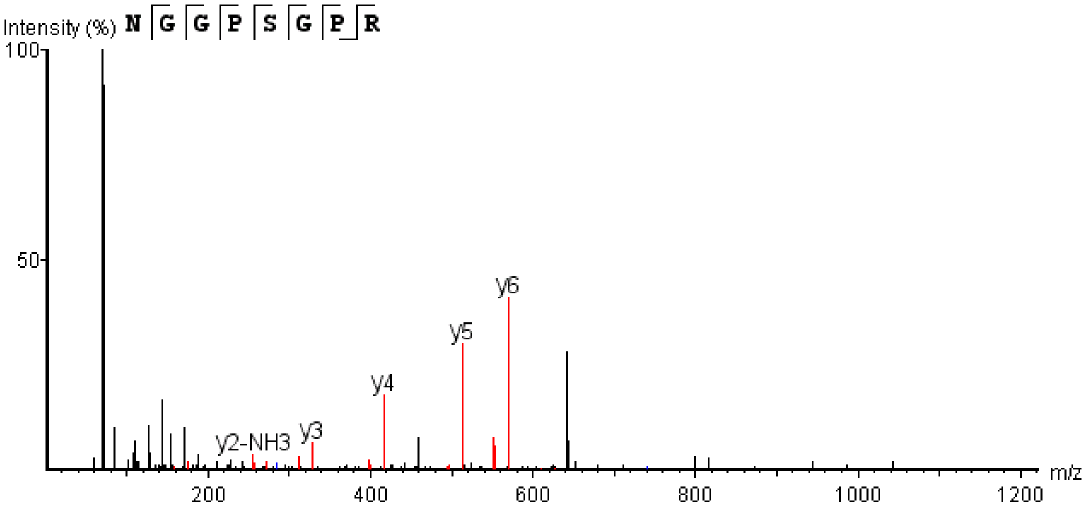

Figure. S2 The result of LC/MS/MS for NGGPSGPR (NGG).

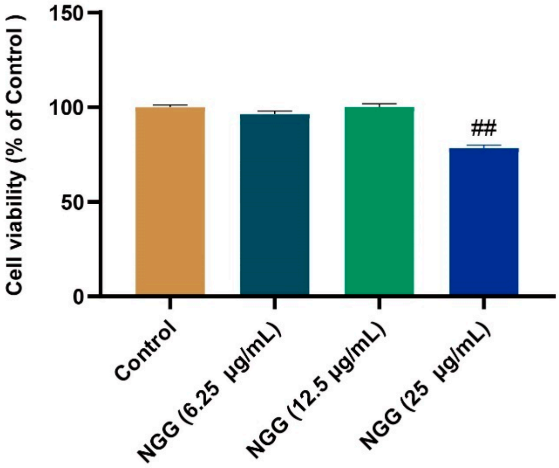

Figure. S3 Results of toxicity of NGG on Human corneal epithelial cells. $n = 5$, $^{##}p < 0.01$, vs control group.

Table S1. Sequence of TSP-II and its prediction by PeptideRanker and ToxinPred.

NO.	Peptide Secquence	Mass (Da)	PeptideRanker Score	ToxinPred
1	GPAGGPLGPR	877.4769	0.899783	Non-Toxin
2	AGPGAGPLGPR	948.514	0.898817	Non-Toxin
3	GPAGPAGPGGPPSGR	1230.6104	0.885754	Non-Toxin
4	AGPRGPAGPSGLR	1191.6472	0.884835	Non-Toxin
5	DFCPPGFNTK	1181.5176	0.88423	Non-Toxin
6	QGPAGGPLGPR	1005.5355	0.880138	Non-Toxin
7	KAPDPF	673.3435	0.867679	Non-Toxin
8	SGPAGPAGPGGPPRQ	1301.6475	0.861916	Non-Toxin
9	QGSGPAGPAGPGGPPQR	1486.7275	0.841318	Non-Toxin
10	SDFPALR	804.413	0.832621	Non-Toxin
11	AGPEGPLGPR	949.4981	0.811454	Non-Toxin

12	AGPSGPAGRP	865.4406	0.807969	Non-Toxin
13	QGGGPQGPVGNTGPKG	1406.6902	0.790962	Non-Toxin
14	GPAGPAGRPGPSGE	1205.5789	0.784207	Non-Toxin
15	VFPGLPDHL	993.5283	0.776531	Non-Toxin
16	GPAGPRGPA	778.4085	0.762096	Non-Toxin
17	PGPSGPKGEPGAK	1177.6091	0.762084	Non-Toxin
18	GPPGTPGSPGPPGFEGGKPQ	1816.8743	0.760928	Non-Toxin
19	NSAGPQGPLGPR	1149.5891	0.751392	Non-Toxin
20	GPAGPRSGPGAA	993.4991	0.742922	Non-Toxin
21	GPSGPRGPA	794.4034	0.742249	Non-Toxin
22	QHGPSGPRGPAGPHGPVGKD	1903.9402	0.740727	Non-Toxin
23	GPRGPAGPHGPVGKD	1397.7163	0.732285	Non-Toxin
24	GPSGRPGPAGPH	1085.5366	0.722768	Non-Toxin
25	AGPAGPRGSPGA	993.4991	0.71577	Non-Toxin
26	AGPRGASGPAGPR	1149.6003	0.70826	Non-Toxin
27	LGPAGPR	666.3813	0.705134	Non-Toxin
28	AGPAGPRGSPGAA	1064.5361	0.704227	Non-Toxin
29	NPSGPGGPTGKDGAGR	1423.6804	0.701498	Non-Toxin
30	NPAGPAGPRGPSGE	1262.6003	0.695935	Non-Toxin
31	FGPGADNAGGK	989.4566	0.690454	Non-Toxin
32	QAGPAGPR	752.3929	0.683607	Non-Toxin
33	GPAGPRGPS	794.4034	0.681857	Non-Toxin
34	SGPAGPRGPSGS	1025.489	0.679261	Non-Toxin
35	KAPDPLRGGHY	1209.6255	0.673412	Non-Toxin
36	AHGPSGPRGPAGPH	1293.6326	0.666935	Non-Toxin
37	QAGPAGPRGSPGAA	1192.5947	0.665539	Non-Toxin
38	QAGPAGPRGSPGA	1121.5576	0.66391	Non-Toxin
39	AAGPSGPRGPA	936.4777	0.66159	Non-Toxin
40	TGGPQGPLGPR	1035.5461	0.661556	Non-Toxin
41	GAHGPSGRPGPAGPH	1350.6541	0.654835	Non-Toxin
42	DFLLK	634.369	0.654617	Non-Toxin
43	GPMGPRGPGPPPGSSGPQ	1644.7678	0.649798	Toxin
44	NGGPSGPR	740.3565	0.648295	Non-Toxin
45	GPAGPRGPSGE	980.4675	0.644932	Non-Toxin
46	LDMLGPR	816.4164	0.643629	Non-Toxin

47	EAPDPLRN	910.4508	0.640178	Non-Toxin
48	GPSGPQGSRGEPGPN	1392.6382	0.63726	Non-Toxin
49	NVGEPGRLF	987.5137	0.627502	Non-Toxin
50	NNPDPLRGGHY	1238.5791	0.626809	Non-Toxin
51	QPPGPTGAP	820.4079	0.620057	Non-Toxin
52	KAPDPLRNHY	1209.6255	0.616423	Non-Toxin
53	QHGGPSPRGPGAGHP	1407.6755	0.613812	Non-Toxin
54	GPAGVRGPAGPA	1005.5355	0.610344	Non-Toxin
55	GPAGPRGSPGSN	1052.4998	0.591846	Non-Toxin
56	PAGGSPRGPA	865.4406	0.582409	Non-Toxin
57	GPAGPRGSPGS	938.457	0.581542	Non-Toxin
58	QPVGEPGKGAGPSGPSGE	1606.7585	0.571822	Non-Toxin
59	EAPDPLRNHY	1210.573	0.554447	Non-Toxin
60	QPGTAAGPGR	910.462	0.552907	Non-Toxin
61	GPVGEPGKQGPSGPSGE	1535.7214	0.550743	Non-Toxin
62	NAPGPGGPTGKDGARQ	1478.7227	0.534334	Non-Toxin
63	QPVGEPGKGAGPSGPS	1420.6946	0.520827	Non-Toxin
64	NPSGPGGPTGKDAGQR	1494.7175	0.505074	Non-Toxin
65	AGPVGEPGKGA	938.4821	0.503477	Non-Toxin
66	VGAGPSGPR	796.4191	0.490303	Non-Toxin
67	TSQFADRL	936.4665	0.484124	Non-Toxin
68	KLPALPAK	836.5483	0.470238	Non-Toxin
69	NPAGPRGPSGE	1037.489	0.469391	Non-Toxin
70	QGGVGELPGR	968.5039	0.469341	Non-Toxin
71	QPTAPDGQPGAK	1165.5728	0.467289	Non-Toxin
72	AGPRGPDSGPSQR	1280.6221	0.465836	Non-Toxin
73	DDVVRF	749.3708	0.464564	Non-Toxin
74	SGLDGAKGDSGPAGPK	1412.6895	0.461318	Non-Toxin
75	FGGRGEPGPAQR	1227.6108	0.458999	Non-Toxin
76	HGPVGKDHSGPGR	1299.6431	0.451958	Non-Toxin
77	GLDGAKGDSGPAGPK	1325.6575	0.442598	Non-Toxin
78	GPVGKDGRGPAH	1146.5894	0.439001	Non-Toxin
79	QNAPGPGGPTGKDQRQ	1606.7812	0.427997	Non-Toxin
80	SGLDGAKGDTGPAGPK	1426.7051	0.427327	Non-Toxin
81	EEAPDPLRNHY	1339.6157	0.41707	Non-Toxin

82	GPVGNTGPK	825.4344	0.4108	Non-Toxin
83	GPHGVPGKD	862.4297	0.409573	Non-Toxin
84	NGVGELPGR	897.4668	0.402994	Non-Toxin
85	LDGAKGDSGPAGPK	1268.636	0.397137	Non-Toxin
86	RGTGPVGMSPGR	1170.5928	0.381551	Non-Toxin
87	DAGKTLPR	856.4766	0.378726	Non-Toxin
88	KGEPGHKGPDRHTSDGPR	1926.9409	0.375891	Non-Toxin
89	DGAKGDTGPAGPK	1169.5676	0.374624	Non-Toxin
90	VRPPAGK	723.4391	0.356249	Non-Toxin
91	RPPAGKD	739.3976	0.355871	Non-Toxin
92	RNGGPEGPAGAR	1137.564	0.352671	Non-Toxin
93	LLAPPER	794.465	0.351403	Non-Toxin
94	LDGAKGDTGPAGPK	1282.6516	0.344193	Non-Toxin
95	VSPDMTSLPTLPK	1400.7222	0.332511	Non-Toxin
96	RNGPEGPAGAR	1080.5425	0.330769	Non-Toxin
97	VKLPALPAK	935.6168	0.319142	Non-Toxin
98	TEPVLPPSKSL	1166.6545	0.317239	Non-Toxin
99	DRGKGEPGAAGPK	1238.6367	0.315499	Non-Toxin
100	LLAPPERKY	1085.6233	0.312105	Non-Toxin
101	EGPKGNRGETGPA	1268.6108	0.307589	Non-Toxin
102	VVRPPAGK	822.5075	0.298109	Non-Toxin
103	AVLLPK	639.4319	0.297171	Non-Toxin
104	SLGGASGTSAMKQ	1209.5659	0.296767	Non-Toxin
105	LEEPGLR	812.4391	0.294498	Non-Toxin
106	VRPAPGKD	838.4661	0.286871	Non-Toxin
107	TGPAGRSPDAGAA	1126.5366	0.286774	Non-Toxin
108	GEEGKRGPTGELG	1285.6262	0.261458	Non-Toxin
109	DVSGGYDEYR	1159.478	0.258236	Non-Toxin
110	DLGDDKVRLS	1116.5774	0.257807	Non-Toxin
111	NPVGNTGPKGA	1010.5145	0.253624	Non-Toxin
112	LDPDTAYK	921.4443	0.250948	Non-Toxin
113	VAPLDHVY	912.4705	0.246187	Non-Toxin
114	LLAPPERKYS	1172.6553	0.239179	Non-Toxin
115	AKDPQSDTGTVGR	1330.6477	0.237518	Non-Toxin
116	TSPVGPTGKP	939.5025	0.234554	Non-Toxin

117	TEAPLNPK	868.4654	0.220992	Non-Toxin
118	VLGLGTR	714.4388	0.20242	Non-Toxin
119	RVRPPAGKD	994.5671	0.195985	Non-Toxin
120	VVRPPAGKD	937.5345	0.195649	Non-Toxin
121	KVLPVGKY	902.5589	0.177888	Non-Toxin
122	LPDTPKE	798.4123	0.172989	Non-Toxin
123	VLLDR	614.3751	0.161797	Non-Toxin
124	LDGYPKPVKEE	1273.6553	0.158243	Non-Toxin
125	LDLVDR	729.4021	0.156631	Non-Toxin
126	SVDDGKDRVS	1076.5098	0.142525	Non-Toxin
127	TDLPKVQ	799.444	0.0882873	Non-Toxin
128	TVPTLPKET	984.5491	0.0878987	Non-Toxin
129	ELTDLPKVQ	1041.5706	0.0848902	Non-Toxin
130	LTGEPSHAV	909.4556	0.0825041	Non-Toxin
131	REVLDSVR	972.5352	0.0754738	Non-Toxin

Table S2. Results of TSP-II screened by ToxinPred and PeptideRanke.

NO.	Peptide	Length	Mass (Da)	ToxinPred	Peptide Ranker Score
1	GPAGGPLGPR	10	877.48	Non-Toxin	0.8998
2	AGPGAGPLGPR	11	948.51	Non-Toxin	0.8988
3	GPAGPAGPGGPPSGR	15	1230.61	Non-Toxin	0.8858
4	AGPRGPAGPSGLR	13	1191.65	Non-Toxin	0.8848
5	DFCPPGFNTK	10	1181.52	Non-Toxin	0.8842
6	QGPAGGPLGPR	11	1005.54	Non-Toxin	0.8801
7	KAPDPF	6	673.34	Non-Toxin	0.8677
8	SGPAGPAGPGGPPRQ	15	1301.65	Non-Toxin	0.8619
9	QGSGPAGPAGPGGPPQR	17	1486.73	Non-Toxin	0.8413
10	SDFPALR	7	804.41	Non-Toxin	0.8326
11	AGPEGPLGPR	10	949.50	Non-Toxin	0.8115
12	AGPSGPAGRP	10	865.44	Non-Toxin	0.8080
13	QGGGPQGPVGNTGPKG	16	1406.69	Non-Toxin	0.7910
14	GPAGPAGRPGPSGE	14	1205.58	Non-Toxin	0.7842
15	VFPGLPDHL	9	993.53	Non-Toxin	0.7765
16	GPAGPRGPA	9	778.41	Non-Toxin	0.7621
17	PGPSGPKGEPGAK	13	1177.61	Non-Toxin	0.7621
18	GPPGTPGSPGPPGFEGGKPQ	20	1816.87	Non-Toxin	0.7609
19	NSAGPQGPLGPR	12	1149.59	Non-Toxin	0.7514
20	GPAGPRSGPGAA	12	993.50	Non-Toxin	0.7429
21	GPSGPRGPA	9	794.40	Non-Toxin	0.7422
22	QHGPSGPRGPAGPHGPVGKD	20	1903.94	Non-Toxin	0.7407

23	GPRGPAGPHGPVGKD	15	1397.72	Non-Toxin	0.7323
24	GPSGRPGPAGPH	12	1085.54	Non-Toxin	0.7228
25	AGPAGPRGSPGA	12	993.50	Non-Toxin	0.7158
26	AGPRGASGPAGPR	13	1149.60	Non-Toxin	0.7083
27	LGPAGPR	7	666.38	Non-Toxin	0.7051
28	AGPAGPRGSPGAA	13	1064.54	Non-Toxin	0.7042
29	NPSGPGGPTGKDGAGR	16	1423.68	Non-Toxin	0.7015
30	NPAGPAGPRGPSGE	14	1262.60	Non-Toxin	0.6959
31	FGPGADNAGGK	11	989.46	Non-Toxin	0.6905
32	QAGPAGPR	8	752.39	Non-Toxin	0.6836
33	GPAGPRGPS	9	794.40	Non-Toxin	0.6819
34	SGPAGPRGPSGS	12	1025.49	Non-Toxin	0.6793
35	KAPDPLRGGHY	11	1209.63	Non-Toxin	0.6734
36	AHGPSGPRGPAGPH	14	1293.63	Non-Toxin	0.6669
37	QAGPAGPRGSPGAA	14	1192.59	Non-Toxin	0.6655
38	QAGPAGPRGSPGA	13	1121.56	Non-Toxin	0.6639
39	AAGPSGPRGPA	11	936.48	Non-Toxin	0.6616
40	TGGPQGPLGPR	11	1035.55	Non-Toxin	0.6616
41	GAHGPSGRPGPAGPH	15	1350.65	Non-Toxin	0.6548
42	DFLLK	5	634.37	Non-Toxin	0.6546
43	NGGPSGPR	8	740.36	Non-Toxin	0.6483
44	GPAGPRGPSGE	11	980.47	Non-Toxin	0.6449
45	LDMLGPR	7	816.42	Non-Toxin	0.6436
46	EAPDPLRN	8	910.45	Non-Toxin	0.6402
47	GPSGPQGSRGEPGPN	15	1392.64	Non-Toxin	0.6373
48	NVGEPGRLF	9	987.51	Non-Toxin	0.6275
49	NNPDPLRGGHY	11	1238.58	Non-Toxin	0.6268
50	QPPGPTGAP	9	820.41	Non-Toxin	0.6201
51	KAPDPLRNHY	10	1209.63	Non-Toxin	0.6164
52	QHGGPSPRGPGAGHP	15	1407.68	Non-Toxin	0.6138
53	GPAGVRGPAGPA	12	1005.54	Non-Toxin	0.6103

Table S3. Results of TSP-II screened by AIPpred and AllerTOP 2.0.

NO.	Peptide	Length	Mass (Da)	AIP or Non-AIP	AIP Score	Allergenicity
1	DFLLK	5	634.369	AIP	0.6023	Yes
2	AGPEGPLGPR	10	949.4981	AIP	0.4767	Yes
3	DFCPPGFNTK	10	1181.518	AIP	0.4651	No
4	NVGEPGRLF	9	987.5137	AIP	0.4581	Yes
5	EAPDPLRN	8	910.4508	AIP	0.4558	No
6	SDFPALR	7	804.413	AIP	0.4465	Yes
7	GPAGPRGPS	9	794.4034	AIP	0.4372	Yes
8	AGPSGPAGRP	10	865.4406	AIP	0.4349	Yes
9	GPAGGPLGPR	10	877.4769	AIP	0.4349	Yes
10	KAPDPLRNHY	10	1209.626	AIP	0.4326	Yes
11	KAPDPF	6	673.3435	AIP	0.4209	Yes

12	LGPAGPR	7	666.3813	AIP	0.4116	Yes
13	GPAGPRGPA	9	778.4085	AIP	0.3977	Yes
14	QAGPAGPR	8	752.3929	AIP	0.3907	Yes
15	GPSGPRGPA	9	794.4034	AIP	0.3791	Yes
16	NGGPSGPR	8	740.3565	AIP	0.3767	No
17	LDMLGPR	7	816.4164	AIP	0.3605	Yes
18	VFPGLPDHL	9	993.5283	Non-AIP	0.3465	Yes
19	QPPGPTGAP	9	820.4079	Non-AIP	0.2512	Yes
